# Ferric carboxymaltose assessment of morbidity and mortality in patients with iron deficiency and chronic heart failure (FAIR‐HF2‐DZHK05) trial: Baseline characteristics and comparison to other relevant clinical trials

**DOI:** 10.1002/ejhf.3658

**Published:** 2025-04-29

**Authors:** Stefan D. Anker, Tim Friede, Javed Butler, Khawaja M. Talha, Marius Placzek, Monika Diek, Anna Nosko, Adriane Stas, Stefan Kluge, Dominik Jarczak, Geraldine Deheer, Meike Rybczynski, Antoni Bayes‐Genis, Frank Edelmann, Gerasimos Filippatos, Gerd Hasenfuß, Wilhelm Haverkamp, Mitja Lainscak, Ulf Landmesser, Iain C. Macdougall, Bela Merkely, Burkert M. Pieske, Fausto J. Pinto, Tienush Rassaf, Maurizio Volterrani, Stephan von Haehling, Markus S. Anker, Wolfram Doehner, Hüseyin Ince, Friedrich Koehler, Gianluigi Savarese, Ursula Rauch‐Kröhnert, Tommaso Gori, Teresa Trenkwalder, Ibrahim Akin, Christina Paitazoglou, Iwona Kobielusz‐Gembala, Witold Zmuda, Luca Kuthi, Norbert Frey, Manuela Licka, Stefan Kääb, Karl‐Ludwig Laugwitz, Piotr Ponikowski, Mahir Karakas

**Affiliations:** ^1^ Deutsches Herzzentrum der Charité, Campus Virchow Klinikum Berlin Germany; ^2^ Institute of Health Center for Regenerative Therapies (BCRT), German Centre for Cardiovascular Research (DZHK) Partner Site Berlin, Charité Universitätsmedizin Berlin Germany; ^3^ Department of Medical Statistics University Medical Center Göttingen Göttingen Germany; ^4^ German Centre for Cardiovascular Research (DZHK), Partner Site Lower Saxony Gottingen Germany; ^5^ Department of Medicine University of Mississippi Medical Center Jackson Mississippi USA; ^6^ Baylor Scott and White Research Institute Dallas Texas USA; ^7^ Department of Cardiovascular Disease Loyola University Medical Center Maywood Illinois USA; ^8^ Department of Intensive Care Medicine University Medical Center Hamburg‐Eppendorf Hamburg Germany; ^9^ Department of Medical Informatics University Medical Center Göttingen Göttingen Germany; ^10^ Department of Cardiology, University Medical Center Hamburg‐Eppendorf University Heart & Vascular Center Hamburg Hamburg Germany; ^11^ German Centre for Cardiovascular Research (DZHK), Partner Site Hamburg/Kiel/Lübeck Hamburg Germany; ^12^ Heart Institute, Hospital Universitari Germans Trias i Pujol, CIBERCV Barcelona Spain; ^13^ Department of Cardiology, Angiology and Intensive Care Medicine Deutsches Herzzentrum der Charité, Campus Virchow Klinikum Berlin Germany; ^14^ German Centre for Cardiovascular Research (DZHK) Partner Site Berlin, Charité Universitätsmedizin Berlin Germany; ^15^ Department of Cardiology Attikon University Hospital, School of Medicine, National and Kapodistrian University of Athens Athens Greece; ^16^ Department of Cardiology and Pneumology, University Medical Centre Göttingen Georg August University of Göttingen Göttingen Germany; ^17^ Division of Cardiology General Hospital Murska Sobota Murska Sobota Slovenia; ^18^ Faculty of Medicine University of Ljubljana Ljubljana Slovenia; ^19^ Berlin Institute of Health Berlin Germany; ^20^ Department of Renal Medicine King's College Hospital London UK; ^21^ Heart and Vascular Center, Semmelweis University Budapest Hungary; ^22^ Division of Cardiology, Department of Internal Medicine University Medicine Rostock Rostock Germany; ^23^ Centro Academico de Medicina de Lisboa, CCUL@RISE, Faculdade de Medicina da Universidade de Lisboa Lisbon Portugal; ^24^ West German Heart‐ and Vascular Center, Department of Cardiology and Vascular Medicine, University Hospital Essen University Duisburg‐Essen Essen Germany; ^25^ IRCCS San Raffaele Roma Rome Italy; ^26^ San Raffaele Open University in Rome Rome Italy; ^27^ School of Cardiovascular and Metabolic Health University of Glasgow Glasgow, Scotland; ^28^ Berlin Institute of Health – Center for Regenerative Therapies, and Department of Cardiology (CVK), Deutsches Herzzentrum der Charité and German Centre for Cardiovascular Research Partner Site Berlin, Charité ‐ Universitätsmedizin Berlin Berlin Germany; ^29^ Deutsches Herzzentrum der Charité (DHZC), Department of Cardiology, Angiology and Intensive Care Medicine, Campus Charité Mitte Berlin Germany; ^30^ Center for Cardiovascular Telemedicine; Charité ‐ Universitätsmedizin Berlin, Corporate Member of Freie Universität Berlin and Humboldt‐Universität zu Berlin Berlin Germany; ^31^ Department of Clinical Science and Education, Södersjukhuset Karolinska Institutet Stockholm Sweden; ^32^ Department of Cardiology, Angiology and Intensive Care Medicine Deutsches Herzzentrum der Charité, Campus Benjamin Franklin Berlin Germany; ^33^ Department of Cardiology, Cardiology I University Medical Center Mainz Mainz Germany; ^34^ German Centre for Cardiovascular Research (DZHK), Standort RheinMain Frankfurt Germany; ^35^ Technical University of Munich, School of Medicine and Health, Department of Cardiovascular Diseases, German Heart Centre Munich, TUM University Hospital Munich Germany; ^36^ German Center for Cardiovascular Research, Partner Site Munich Heart Alliance Munich Germany; ^37^ Department of Cardiology, Angiology, Haemostaseology and Medical Intensive Care University Medical Center Mannheim, Medical Faculty Mannheim, Heidelberg University Heidelberg Germany; ^38^ DZHK (German Center for Cardiovascular Research (DZHK)), Partner Site, Heidelberg/Mannheim Mannheim Germany; ^39^ Department of Cardiology, Angiology and Intensive Care Medicine, University Heart Center Lübeck, Medical Clinic II University Hospital Schleswig‐Holstein Lübeck Germany; ^40^ Oświęcimskie Centrum Badań Klinicznych Oswiecim Poland; ^41^ Department of Cardiology, Angiology and Pneumolgy, Clinical Trial Unit University Hospital Heidelberg Heidelberg Germany; ^42^ LMU Munich, Department of Medicine I LMU University Hospital Munich Germany; ^43^ Department of Internal Medicine I Technical University Munich University Hospital Munich Germany; ^44^ Institute of Heart Disease, Medical University and University Hospitals Wroclaw Poland

**Keywords:** Heart failure, Iron deficiency, Clinical trial, Hospitalization, Transferrin saturation

## Abstract

**Aims:**

Prior randomized trials have reported conflicting evidence regarding the efficacy of intravenous (IV) iron in patients with heart failure with reduced ejection fraction (HFrEF) and iron deficiency (ID).

**Methods and results:**

FAIR‐HF2 is a double‐blind, randomized, controlled trial evaluating the efficacy of IV ferric carboxymaltose in patients with HFrEF and ID. We report the baseline characteristics of enrolled patients and compare them with other major trials of IV iron in HFrEF (FAIR‐HF, CONFIRM‐HF, AFFIRM‐AHF, IRONMAN, and HEART‐FID). A total of 1105 patients were randomized between March 2017 and November 2023. Most patients were men (67%) and median age was 72 (interquartile range [IQR] 63–79) years. More than one‐third had a heart failure hospitalization within the preceding 12 months (36%), and 53% were hospitalized at randomization. Common comorbidities included hypertension (79%), coronary artery disease (74%), dyslipidaemia (67%), and diabetes (46%). The median left ventricular ejection fraction was 58% (IQR 42–77) and mean estimated glomerular filtration rate was 58 (IQR 42–77) ml/min/1.73 m^2^. A total of 1064 (96%) patients were on renin–angiotensin system inhibitors (angiotensin receptor–neprilysin inhibitors [ARNI] 38%), 1016 (92%) on beta‐blockers, and 779 (71%) on mineralocorticoid receptor antagonists; and 261 (24%) of patients were on sodium–glucose cotransporter 2 (SGLT2) inhibitors, which is much higher than prior trials. A higher proportion of patients had ischaemic HFrEF (78%) compared to preceding trials. The baseline median haemoglobin (g/dl) was 12.7 (IQR 11.8–13.4), median serum ferritin (μg/dl) was 63 (IQR 36–90), and median transferrin saturation (%) was 16.5 (IQR 11.8–22.9), resembling that of other trials. The mean 6‐min walk distance at enrolment was 314 ± 118 m.

**Conclusion:**

The FAIR‐HF2 trial represents a contemporary cohort of patients with baseline characteristics mostly similar to prior trial populations. Use of SGLT2 inhibitors and ARNI in FAIR‐HF2 was higher than in prior trials.

Clinical Trial Registration: ClinicalTrials.gov ID NCT03036462.

## Introduction

Several modest to large scale clinical trials have evaluated the role of intravenous (IV) iron on outcomes in patients with heart failure (HF) with reduced ejection fraction (HFrEF) with concomitant iron deficiency (ID). Although earlier trials suggested a consistent benefit of IV iron in improving symptoms and functional status, and reducing HF hospitalizations in HFrEF,^1^, ^2^, ^3^ more recent trials have been unable to replicate the same level of treatment benefit in larger cohorts of patients with chronic stable HFrEF.^4^, ^5^ These differences can be attributed to differences in IV iron dosing strategies, a lack of consistent IV iron administration during the maintenance phase following repletion of iron stores, slow enrolment and low adherence to therapy during the COVID‐19 pandemic, low utilization of sodium–glucose cotransporter 2 (SGLT2) inhibitors, and the questionable appropriateness of the contemporary criteria for diagnosing ID in HFrEF.^6^, ^7^, ^8^

The FAIR‐HF2 (Ferric carboxymaltose assessment of morbidity and mortality in patients with iron deficiency and chronic heart failure) trial is designed to evaluate the efficacy of a more intensive and proactive regimen of IV iron (i.e. ferric carboxymaltose [FCM]) on clinical outcomes in patients with HFrEF. Herein, we describe the baseline characteristics of the FAIR‐HF2 trial and compare them to baseline characteristics of patients enrolled in prior major trials of IV iron in HFrEF.

## Methods

The design of the FAIR‐HF2 trial has been described previously.^9^ Briefly, it is an international, double‐blind, multicentre randomized controlled trial evaluating the efficacy of FCM versus placebo in patients with chronic HFrEF (left ventricular ejection fraction [LVEF] ≤45%) with concomitant ID. All patients were at least 18 years of age, have chronic HFrEF for at least 3 months with a documented LVEF ≤45%. Patients were also required to be eligible for discharge within 24 h if hospitalized with New York Heart Association (NYHA) class II or III symptoms, or ambulatory with NYHA class II–IV symptoms with a hospitalization for HF within the past 12 months. Key laboratory criteria for ID included a haemoglobin between 9.0 and 14.0 g/dl (in both, women and men), and the confirmed presence of ID defined as serum ferritin <100 ng/ml or between 100 ng/ml and 299 ng/ml (i.e. <300 ng/ml), if the transferrin saturation (TSAT) is <20%. The iron repletion protocol consisted of two phases; the repletion phase involved administration of 1000 to 2000 mg of FCM over 4 weeks followed by a maintenance phase which involved administration of 500 mg of FCM every 4 months until study end, with stopping criteria of haemoglobin >16 g/dl or serum ferritin >800 ng/ml. The trial has three co‐primary endpoints: (i) time to first event of cardiovascular death or hospitalization for HF, (ii) the rate of total (first and recurrent) HF hospitalizations (both analysed in the full study population), and (iii) the time to first event of cardiovascular death or hospitalization for HF in patients with TSAT <20% at baseline.^10^

### Baseline data

All patients underwent a baseline visit following enrolment in which medical and social history was obtained through chart review and individual interviews with patients. Variables collected include history of hypertension, prior myocardial infarction, stroke, hyperlipidaemia, atrial fibrillation/flutter, type 2 diabetes, HFrEF aetiology, and smoking history. Background therapy for HFrEF including angiotensin‐converting enzyme inhibitors (ACEi)/angiotensin receptor blockers (ARB)/angiotensin receptor–neprilysin inhibitor (ARNI), beta‐blockers, mineralocorticoid receptor antagonists, SGLT2 inhibitors, loop diuretics, and implantable cardioverter‐defibrillator/cardiac resynchronization therapy (ICD/CRT) were recorded at baseline visit. Laboratory data include serum ferritin, serum iron, serum transferrin, TSAT, and estimated glomerular filtration rate (eGFR). The 6‐min walk test and the EuroQol‐5 dimensions (EQ‐5D) assessment were performed at the baseline visit. For continuous data, means with standard deviation and median with interquartile range (IQR) were calculated as appropriate. For categorical data, frequencies (in %) are provided.

### Comparison with other major intravenous iron trials in heart failure

In this study, a comparison between major baseline characteristics is provided between FAIR‐HF2 and five prior major IV iron trials (FAIR‐HF, CONFIRM‐HF, AFFIRM‐AHF, IRONMAN, and HEART‐FID).^1^, ^2^, ^3^, ^4^, ^5^

Briefly, the FAIR‐HF evaluated the efficacy of IV FCM in patients with HFrEF (NYHA class II with LVEF ≤40% or NYHA class III with LVEF ≤45%) and ID, defined as serum ferritin <100 ng/ml or 100 ng/ml to 300 ng/ml and TSAT <20%. The two primary endpoints were self‐reported Patient Global Assessment (PGA) and changes in NYHA functional status at 24 weeks. Patients in the treatment arm had a significantly improved PGA and NYHA functional status (odds ratio +2.51; 95% confidence interval [CI] 1.75–3.61 and odds ratio for improvement by one class 2.40; 95% CI 1.55–3.71, respectively) at 24 weeks.^1^

The CONFIRM‐HF evaluated the efficacy of IV FCM in 304 patients with stable, ambulatory HFrEF (LVEF ≤45%) and ID, defined as serum ferritin <100 ng/ml or 100 to 300 ng/ml and TSAT <20%. The primary endpoint was the change in 6‐min walk distance at 24 weeks from baseline. Patients in the treatment arm had a significant improvement in the primary endpoint (difference FCM vs. placebo: 33 ± 11 m, *p* = 0.002). The secondary endpoint of hospitalization for HF also occurred less frequently among patients in the treatment arm (hazard ratio 0.39; 95% CI 0.19–0.82, *p* = 0.009).^2^

The AFFIRM‐AHF trial evaluated the efficacy of FCM in 1132 patients with recent worsening HFrEF (LVEF ≤50%) with ID defined as a serum ferritin of <100 μg/L, or 100 to 299 μg/L with TSAT <20%. The primary endpoint was a composite of HF hospitalizations and cardiovascular death for up to 1 year. The trial did not meet its primary endpoint (rate ratio 0.79; 95% CI 0.62–1.01, *p* = 0.059), but found a significant reduction in total HF hospitalizations (rate ratio 0.67; 95% CI 0.47–0.97, *p* = 0.035).^3^

The IRONMAN trial evaluated the efficacy of IV ferric derisomaltose in 1137 patients with HFrEF (LVEF ≤45%) with ID defined as serum ferritin of <100 μg/L or a TSAT <20%. The primary endpoint was the composite of all‐cause death and recurrent HF hospitalizations. The trial did not meet its primary endpoint (rate ratio 0.82; 95% CI 0.66–1.02, *p* = 0.07), but the trial was significantly affected by the COVID‐19 pandemic. A sensitivity analysis for the COVID‐19 pandemic showed a significant reduction in the primary endpoint favouring ferric derisomaltose (rate ratio 0.76; 95% CI 0.58–1.00, *p* = 0.047).^4^

The HEART‐FID trial is the largest IV iron trial to date. It evaluated the efficacy of IV FCM in 3065 patients with chronic, stable HFrEF (LVEF ≤40%) and ID, defined as haemoglobin level >9.0 g/dl and <13.5 g/dl in women (<15.0 g/dl in men), and serum ferritin <100 ng/ml or 100 to 300 ng/ml with TSAT <20%. The primary outcome was a hierarchical composite of death and HF hospitalizations within 12 months after randomization or change in the 6‐min walk distance from baseline. The trial did not meet its primary endpoint with no significant difference between the IV FCM and placebo groups (win ratio 1.10; 95% CI 0.99–1.23). The mean change in 6‐min walk distance favoured the FCM group (8 ± 60 vs. 4 ± 59 m, *p* = 0.02), but the difference between groups was very small.^5^

## Results

A total of 1105 patients were randomized between March 2017 and November 2023 in 54 active sites from six countries. Baseline relevant clinical characteristics and HF‐related characteristics are provided in *Tables* *1* and *2*. The majority of patients were men (67%) and the median age was 72 (IQR 63–79). The median body mass index (kg/m^2^) was 27.5 (IQR 24.1–31.3). Most patients had either NYHA class II (66%) or class III symptoms (32%). Almost half the patients had diabetes mellitus (46%), atrial fibrillation/flutter (52%), and a prior myocardial infarction (48%); 79% had hypertension, 74% had coronary artery disease, and 67% had dyslipidaemia. More than one‐third of patients (402 [36.4%]) had a hospitalization within the prior 12 months before enrolment. The median LVEF (%) was 35 (IQR 28–40), and the median eGFR was 58 (IQR 42–77) ml/min/1.73 m^2^.

**Table 1:** Relevant baseline characteristics of the FAIR‐HF2 population

Patients, *n*	1105
Women, *n* (%)	368 (33)
Age (years), median (IQR)	72 (63–79)
Race/ethnicity, *n* (%)	
White	1090 (99)
Black	7 (0.6)
Past medical history, *n* (%)	
Diabetes mellitus	503 (46)
Hypertension	872 (79)
Hyperlipidaemia	744 (67)
Smoking history	561 (51)
Coronary heart disease	813 (74)
Prior myocardial infarction	526 (48)
Atrial fibrillation/flutter	572 (52)
Stroke/transient ischaemic attack	177 (16)
Chronic obstructive pulmonary disease	192 (17)
Congenital heart disease	24 (2)
Prior percutaneous coronary intervention	568 (51)
Prior coronary artery bypass graft	207 (19)
Physical examination findings, *n* (%)	
Dyspnoea on exertion	1024 (93)
Dyspnoea at rest	59 (5)
Peripheral oedema	478 (43)
Jugular venous distention	42 (4)
Pulmonary rales	277 (25)
Vitals, median (IQR)	
Weight (kg)	81 (70–94)
Body mass index (kg/m^2^)	27 (24–31)
Systolic BP (mmHg)	120 (107–132)
Diastolic BP (mmHg)	71 (64–80)
Heart rate (bpm)	69 (62–76)
Laboratory variables	
Haemoglobin (g/dl), median (IQR)	12.7 (11.8–13.4)
Ferritin (μg/L), median (IQR)	63 (36–90)
30 μg/L≤ferritin≤100 μg/L, *n* (%)	696 (63)
Ferritin >100 μg/L, *n* (%)	191 (17)
Ferritin <30 μg/L, *n* (%)	218 (20)
Iron (μmol/L), median (IQR)	10.8 (7.9–14.9)
Iron ≤13 μmol/L, *n* (%)	705 (65)
Transferrin (mg/dl), median (IQR)	261 (231–297)
Transferrin saturation (%), median (IQR)	16.5 (11.8–22.9)
Transferrin saturation <20%, *n* (%)	748 (68)
Anaemia (WHO)^a^, *n* (%)	526 (48)
eGFR (CKD‐EPI) (ml/min/1.73 m^2^), median (IQR)	58 (42–77)
Quality of life assessment	
6‐min walk distance, mean (± SD)	314 (118)
6‐min walk distance, median (IQR)	323 (232–399)
EQ‐5D, mean (± SD)	0.82 (0.20)
EQ‐5D, median (IQR)	0.89 (0.77–1.00)

BP, blood pressure; CKD‐EPI, Chronic Kidney Disease Epidemiology Collaboration; eGFR, estimated glomerular filtration rate; EQ‐5D, EuroQol‐5 dimensions; IQR, interquartile range; SD, standard deviation; WHO, World Health Organization.

^a^ WHO definition of anaemia: haemoglobin <13 g/dl for men and <12 g/dl for women.

**Table 2:** Heart failure‐related clinical characteristics

Patients, *n*	1105
Ischaemic aetiology, *n* (%)	858 (78)
NT‐proBNP (pg/ml), median (IQR)	
Overall	1895 (839–4853)
Patients with a history of atrial fibrillation/flutter or documented presence of atrial fibrillation/flutter on ECG at baseline (N=578)	2954 (1200–5543)
Patients without a history of atrial fibrillation/flutter or without a documented presence of atrial fibrillation/flutter on ECG at baseline (N=527)	1152 (588–3005)
NYHA functional class, *n* (%)	
I	10 (1)
II	734 (66)
III	356 (32)
IV	5 (0.5)
Heart failure therapy, *n* (%)	
Angiotensin‐converting enzyme inhibitor	455 (41)
Angiotensin receptor blocker	190 (17)
Angiotensin receptor blocker	419 (38)
Beta‐blocker	1016 (92)
Mineralocorticoid receptor antagonist	779 (7)
Sodium–glucose cotransporter 2 inhibitor	261 (24)
Diuretics	906 (82)
ICD/CRT	546 (49)
Prior hospitalization within the prior 12 months, *n* (%)	402 (36)

CRT, cardiac resynchronization therapy; ICD, implantable cardioverter‐defibrillator; IQR, interquartile range; NT‐proBNP, N‐terminal pro‐B‐type natriuretic peptide; NYHA, New York Heart Association.

In FAIR‐HF2, 78% of patients had ischaemic HFrEF which is similar to that observed in FAIR‐HF (80%), whereas 57% patients in the IRONMAN trial, 60% in the HEART FID trial, and 47% in the AFFIRM‐AHF trial were deemed to have ischaemic HFrEF. The mean LVEF was similar across the five trials. The distribution of other comorbidities was similar as shown in *Table* *3*.

**Table 3:** Comparison of baseline characteristics between the FAIR‐HF, CONFIRM‐HF, AFFIRM‐AHF, IRONMAN, HEART‐FID, and FAIR‐HF2 trials

	FAIR‐HF	CONFIRM‐HF	AFFIRM‐AHF	IRONMAN	HEART‐FID	FAIR‐HF2
*n*	459	301	1132	1137	3065	1105
Age (years), mean	68	69	71.0	73	70	70
Women, *n* (%)	244 (53)	141 (47)	488 (44%)	300 (26)	1037 (34%)	368 (33%)
Race, *n* (%)						
White	458 (100)	299 (99)	1051 (95)	1043 (92)	2649 (86)	1090 (99)
Past medical history, *n* (%)						
Ischaemic aetiology	368 (80)	251 (83)	522 (47)	647 (57)	1837 (60)	858 (78)
Prior myocardial infarction	258 (56)	180 (60)	442 (40)	577 (51)	1423 (56)	526 (48)
Stroke	33 (7)	45 (15)	119 (11)	NR	359 (12)	177 (16)
Hypertension	371 (81)	360 (86)	939 (85)	612 (54)	2608 (85)	872 (79)
Atrial flutter/fibrillation	138 (30)	139 (46)	619 (56)	534 (47)	1330 (48)	572 (52)
Hyperlipidaemia	214 (47)	196 (65)	592 (53)	NR	1979 (65)	744 (67)
Type 2 diabetes	130 (28)	83 (27.6)	454 (41)	521 (46)	1385 (45)	503 (46)
Smoking history	NR	95 (32)	419 (38)	NR	1481 (48)	561 (51)
NYHA functional class, *n* (%)						
II	82 (18)	171 (57)	495 (45)	648 (57)	1617 (53)	734 (66)
III	377 (82)	130 (43)	549 (50)	468 (41)	1403 (46)	356 (32)
LVEF (%), mean	32	37	33	34	32	34
Vitals, mean						
Systolic BP (mmHg)	126	125	120	119	123	119
Diastolic BP (mmHg)	77	75	72	NR	74	71
Heart rate (bpm)	71	70	74	NR	73	70
Body mass index (kg/m^2^)	28.1	28.0	28.1	28.4	28.5	28.2
Laboratory variables						
Haemoglobin (g/dl), mean	11.9	12.4	12.2	12.1	12.6	12.5
Serum ferritin (μg/L)^b^, mean	55.1	57.1	86.2	49.5 (median)	56.7	73.0 (median 62.7)
Transferrin saturation (%)^b^, mean	17.4	19.2	14.7	15.0 (median)	23.5	18.3 (median 17.0)
Transferrin saturation <20%, *n* (%)	NR	190 (63)	926 (84)	841 (74)	1324 (43)	748 (68)
NT‐proBNP (pg/ml)	NR	2511 (mean)	4743 (median)	NR	1462 (median)	1895^a^ (median)
Background medical therapy, *n* (%)						
ACEi/ARB/ARNI	422 (92)	305 (100)	844 (76)	984 (86)	2733 (86)	1064 (96)
ARNI	NR	NR	NR	240 (21)	909 (30)	419 (38)
Beta‐blocker	391 (85)	272 (90)	914 (82)	1009 (89)	2833 (92)	1016 (92)
MRA	NR	NR	728 (66)	632 (56)	1705 (56)	779 (70)
SGLT2 inhibitor	NR	NR	NR	29 (3)	229 (8)	261 (24)
ICD/CRT‐D	NR	NR	194 (18)	407 (36)	1441 (47)	546 (49)

ACEi, angiotensin‐converting enzyme inhibitor; ARB, angiotensin receptor blocker; ARNI, angiotensin receptor–neprilysin inhibitor; BMI, body mass index; BP, blood pressure; CRT‐D, cardiac resynchronization therapy with defibrillator; ICD, implantable cardioverter‐defibrillator; LVEF, left ventricular ejection fraction; MRA, mineralocorticoid receptor antagonist; NR, not reported; NT‐proBNP, N‐terminal pro‐B‐type natriuretic peptide; NYHA, New York Heart Association; SGLT2, sodium–glucose contransporter 2.

^a^ 728 patients had NT‐proBNP values reported in the FAIR‐HF2 trial.

^b^ Averaged median of both treatment groups provided for IRONMAN. Averaged mean of both treatment groups provided for HEART‐FID.

### Background heart failure therapy

A total of 1064 (96.2%) patients were on renin‐angiotensin system inhibitors, 1016 (91.9%) patients were on beta‐blockers, and 779 (70.5%) patients were on mineralocorticoid receptor antagonists. Almost a quarter of patients (261 [23.6%]) were on SGLT2 inhibitors, and 906 (82%) patients were taking diuretics (*Table* *2*).

The use of guideline‐directed HFrEF therapies is largely similar across the FAIR‐HF, CONFIRM‐HF, IRONMAN, HEART‐FID, and FAIR‐HF trials, except for SGLT2 inhibitors, the use of which was much more prevalent (24%) in FAIR‐HF2 compared to IRONMAN (<3%) and HEART‐FID (7.7%), and ARNI (38%) compared to IRONMAN (21%) and HEART‐FID (30%) (*Table* *3*).

### Laboratory evaluation

The baseline median haemoglobin (g/dl) was 12.7 (IQR 11.8–13.4), median serum ferritin (μg/dl) was 63 (IQR 36–90), and median transferrin saturation (%) was 16.5 (IQR 11.8–22.9) (*Table* *1*).

The mean TSAT is similar to that in the FAIR‐HF (18.3%), CONFIRM‐HF, AFFIRM‐AHF, and IRONMAN trials but lower than that amongst patients in the HEART‐FID trial (24%).^11^ Baseline serum ferritin levels are higher in the FAIR‐HF2 trial compared to that in the IRONMAN (mean 50 μg/dl) and HEART‐FID (mean 57 μg/dl), but lower than that in the AFFIRM‐AHF trial (mean 86 μg/dl) (*Table* *3*).

### Functional parameters

The mean 6‐min walk distance at enrolment was 314 ± 118 m (median 323 m [IQR 232–399]) and the mean EQ‐5D score was 0.82 ± 0.20 (median 0.89 [IQR 0.77–1.00]) (*Table* *1*). The mean 6‐min walk distance was numerically higher in the FAIR‐HF2 trial compared to the CONFIRM‐HF (288–302 m) and IRONMAN trials (286 m).

## Discussion

We present details pertaining to the baseline characteristics of patients included in the FAIR‐HF2 trial in comparison to past major IV iron trials in HFrEF and we present several key findings. The mean %TSAT is less than that observed in the HEART‐FID trial, but largely similar to what was observed in the other four trials. There is also a much higher proportion of patients with ischaemic HFrEF, and those using SGLT2 inhibitors compared to prior trials (*Graphical Abstract*).

**Figure 1:**
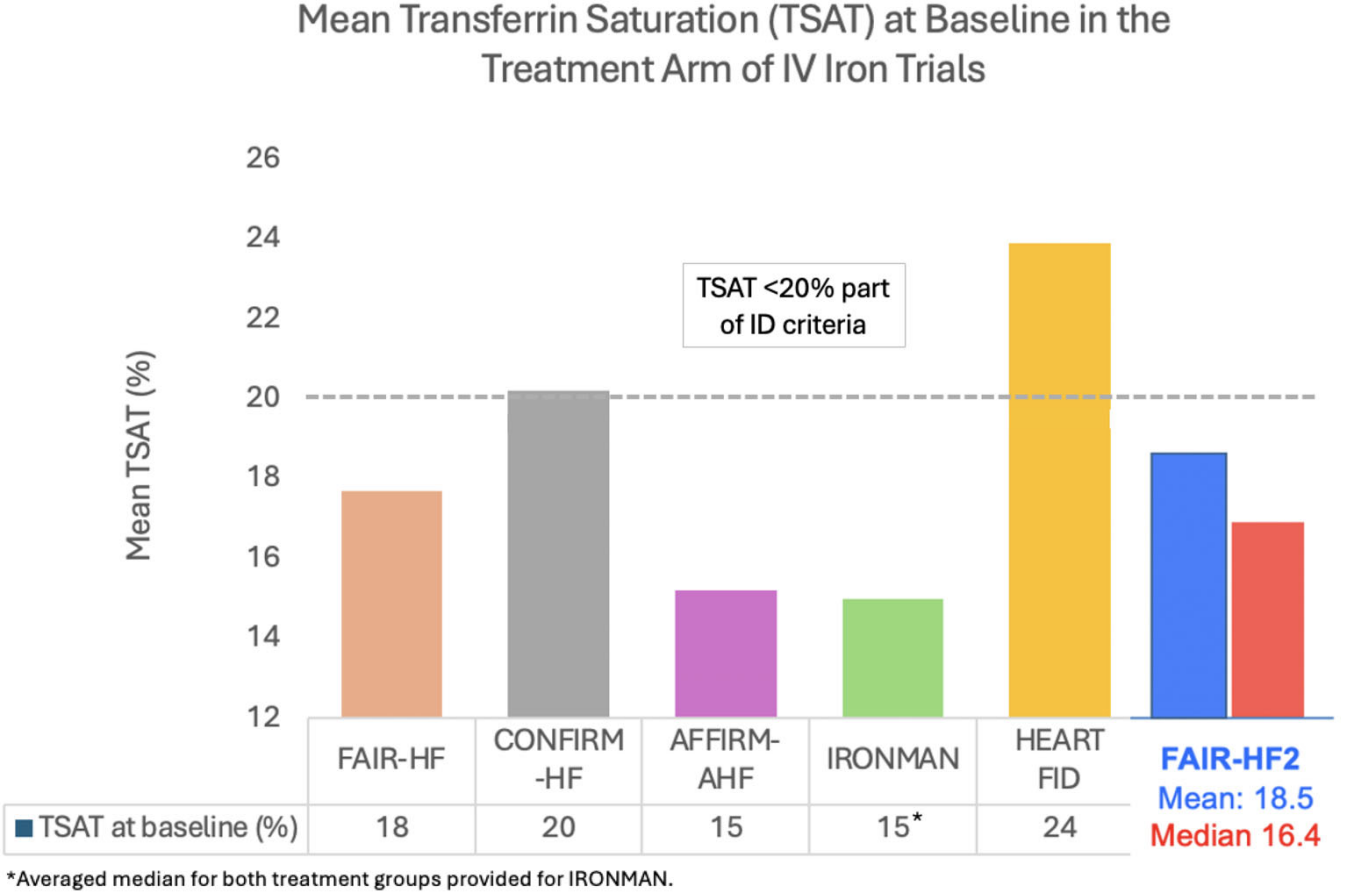
Comparison of baseline mean transferrin saturation percentage between the FAIR‐HF, CONFIRM‐HF, AFFIRM‐AHF, IRONMAN, HEART‐FID, and FAIR‐HF2 trials. Adapted from Talha *et al*.^6^

That the FAIR‐HF2 population had relatively low %TSAT values at baseline (*Figure* *1*) is important as lower values provide more certainty that truly iron‐deficient patients were included. The recently suggested new and more simple definition of ID in HF relies on a TSAT cut‐off of <20%. The rather high mean %TSAT of 22% at baseline amongst patients in the HEART‐FID trial (median 23–24%),^5^ suggests that a substantial proportion of patients there may not have been truly deficient which may explain the suboptimal results found there. The FAIR‐HF2 will include a primary endpoint that will compare the efficacy of IV iron in patients with TSAT <20% versus those with TSAT ≥20%. Evaluating the efficacy of IV iron is challenging due to several reasons. First, the criteria for defining ID have been scrutinized, with evidence suggesting little role of serum ferritin in defining ID, with %TSAT playing a much more central role, specifically in the identification of patients with functional ID.^7^, ^12^, ^13^, ^14^ This is important since accurate identification of ID would help recruit individuals that would potentially benefit most from IV iron repletion. Second, unlike other drugs used in HF, IV iron therapy has two phases to its administration which includes the initial repletion phase after initial diagnosis, followed by a maintenance phase after iron stores have been repleted.^6^ The dosing regimen for both these phases has not been standardized, especially for the latter where most patients enrolled in prior clinical trials did not receive any IV iron during the maintenance phase.^6^, ^15^ In the HEART‐FID trial, only 20% of patients received IV FCM during the maintenance phase. The FAIR‐HF employed a more aggressive approach toward repletion and maintenance of iron stores with an initial regimen of FCM 1000–2000 mg given during the repletion phase followed by FCM 500 mg given every 4 months to prevent transient depletion of iron stores, which may have affected results of prior studies.

Sodium–glucose cotransporter 2 inhibitors are the latest addition to guideline‐directed medical therapy that improves outcomes in patients with HFrEF.^16^, ^17^, ^18^ These drugs have also been shown to augment the haematological response to IV iron supplementation in patients with HFrEF.^8^, ^19^ On the other hand, they have also been shown to reduce levels of serum ferritin attributed largely to their anti‐inflammatory effect rather than an effect on actual iron stores.^19^, ^20^ Given that the addition of SGLT2 to medical therapy regimen in HFrEF is very recent, prior trials have not had a sufficiently large number of patients on SGLT2 inhibitors, precluding an analysis of their effect on the efficacy of IV iron. Similarly, neprilysin inhibition is also associated with suppression of serum ferritin likely due to its anti‐inflammatory effect similar to SGLT2 inhibitors.^13^ In FAIR‐HF2, 23% of patients were using SGLT2 inhibitors and 38% were using ARNI therapy, which is still not optimal, but is likely sufficient for an exploratory analysis. On the contrary, a larger proportion of patients were on optimal guideline‐directed medical therapy in FAIR‐HF2 which may lead to lower cardiovascular events compared to prior trials.^21^ Moreover, a large proportion of patients in the FAIR‐HF2 had ischaemic aetiology for their HFrEF (78%), which is similar to that observed in the FAIR‐HF trial, whereas a much lower proportion of patients had ischaemic HFrEF in the AFFIRM‐AHF, IRONMAN, and HEART‐FID trials.^1^, ^4^, ^5^ Individuals with ischaemic HFrEF have significantly worse prognosis compared to those with non‐ischaemic HFrEF and potentially derive a greater benefit from administration of IV iron.^22^, ^23^ Iron may in a beneficial way support cardiac metabolism that is deranged in HF, and possibly particularly so in patients with ischaemic disease aetiology.^23^

The FAIR‐HF2 trial recruited patients from Western and Eastern Europe with a predominantly White population included in the study, so the results of the study may not be globally generalizable to individuals of other races/ethnicities,^24^ although there are limited data regarding variation in response to IV iron therapy based on patients' race/ethnicity. Most other demographics, including the proportion of women and clinical comorbidities, were similar across the four major IV iron trials.

In conclusion, the FAIR‐HF2 trial is evaluating the efficacy of IV iron in patients with HFrEF and concomitant ID, and the baseline characteristics of enrolled participants largely resemble that of prior major IV iron trials except for a higher proportion of patients with ischaemic HFrEF and those using SGLT2 inhibitors and ARNI therapy.

## Supporting information


**Appendix S1.** Supporting Information.
