# Supplementary material for: Ferric carboxymaltose assessment of morbidity and mortality in patients with iron deficiency and chronic heart failure (FAIR‐HF2‐DZHK05) trial: Baseline characteristics and comparison to other relevant clinical trials
Source: Eur J Heart Fail. 2025 Apr 29;27(8):1436–43. doi: 10.1002/ejhf.3658 (PMC12482851; doi:10.1002/ejhf.3658)
Supplement: Supplementary file 1 — Appendix S1. Supporting Information. [file EJHF-27-1436-s001.docx]

**FINANCIAL DISCLOSURES**

**Stefan D. Anker** reports grants and personal fees from Vifor and Abbott Vascular, and personal fees for consultancies, trial committee work and/or lectures from Actimed, Astra Zeneca, Bayer, Bioventrix, Boehringer Ingelheim, Brahms, Cardiac Dimensions, Cardior, Cordio, CVRx, Cytokinetics, Edwards, Farraday Pharmaceuticals, GSK, HeartKinetics, Impulse Dynamics, Medtronic, Novartis, Novo Nordisk, Occlutech, Pfizer, Regeneron, Relaxera, Repairon, Scirent, Sensible Medical, Servier, Vectorious, and V-Wave. Named co-inventor of two patent applications regarding MR-proANP (DE 102007010834 & DE 102007022367), but he does not benefit personally from the related issued patents.

**Tim Friede** reports payments to institution from Abbott, grants from Deutsche Forschungsgemeinschaft (DFG), Federal Joint Committee (G-BA) and European Commission; consulting fees from Actimed, Bayer, BMS, CSLBehring, Daiichi Sankyo, Galapagos, Immunic, KyowaKirin, LivaNova, Minoryx, Novartis, RECARDIO, Relaxera, Roche, Servier, Viatris, and Vifor, payments from Fresenius Kabi and PINK gegen Brustkrebs, is Trial Data Monitoring Committee member of Aslan, Bayer, BiosenseWebster, Enanta, Galapagos, IQVIA, Novartis, PPD, Recordati, Roche, VICO Therapeutics and is Trial Steering Committee member of SCLBehring.

**Javed Butler** reports consulting fees from Abbott, American Regent, Amgen, Applied Therapeutic, AskBio, Astellas, AstraZeneca, Bayer, Boehringer Ingelheim, Boston Scientific, Bristol Myers Squibb, Cardiac Dimension, Cardiocell, Cardior, Cardiorem, CSL Bearing, CVRx, Cytokinetics, Daxor, Edwards, Element Science, Faraday, Foundry, G3P, Innolife, Impulse Dynamics, Imbria, Inventiva, Ionis, Lexicon, Lilly, LivaNova, Janssen, Medtronics, Merck, Occlutech, Owkin, Novartis, Novo Nordisk, Pfizer, Pharmacosmos, Pharmain, Pfize, Prolaio, Regeneron, Renibus, Roche, Salamandra, Sanofi, SC Pharma, Secretome, Sequana, SQ Innovation, Tenex, Tricog, Ultromics, Vifor, and Zoll, and honoraria from Novartis, Boehringer Ingelheim-Lilly, Astra Zeneca, Impulse Dynamics, Vifor.

**Khawaja M. Talha** reported no conflict of interest.

**Marius Placzek** reported no conflict of interest.

**Monika Diek** reported no conflict of interest.

**Anna Nosko** reported no conflict of interest.

**Adriane Stas** reported no conflict of interest.

**Stefan Kluge** received research support from Cytosorbents and Daiichi Sankyo. He also received lecture fees from ADVITOS, Biotest, CSL Behring, Daiichi Sankyo, Fresenius Medical Care, Gilead, Mitsubishi Tanabe Pharma, MSD, Pfizer, Shionogi and Zoll. He received consultant fees from ADVITOS, Fresenius, Gilead, MSD and Pfizer.

**Dominik Jarczak** reported no conflict of interest.

**Geraldine deHeer** reported no conflict of interest.

**Meike Rybczynski** reported no conflict of interest.

**Antoni Bayes-Genis** has lectured and/or participated in advisory boards for Abbott, AstraZeneca, Bayer, Boehringer-Ingelheim, Medtronic, Novartis, Novo Nordisk, Roche Diagnostics, Vifor.

**Frank Edelmann** reported no conflict of interest.

**Gerasimos Filippatos** has participated in committees for trials and registries sponsored by Novartis, Servier, Medtronic, Vifor, Boehringer Ingelheim, and Bayer.

**Gerd Hasenfuß** reported no conflict of interest.

**Wilhelm Haverkamp** reported no conflict of interest.

**Mitja Lainscak** reports grant from Slovenian Research Agency and honoraria from Novartis, Boehringer Ingelheim and AstraZeneca.

**Ulf Landmesser** reports grants from Abbott and Novartis and consulting fees and honoraria from Abbott.

**Iain C. Macdougall** reports consulting fees from GlaxoSmithKline and Vifor Pharma.

**Bela Merkely** has received speaker fees and/or research payments from Abbott, AstraZeneca, Biotronik, Boehringer Ingelheim, CSL Behring, Daiichi-Sankyo, DUKE Clinical Institute, Medtronic, and Novartis; and has received institutional grants from Abbott, AstraZeneca, Biotronik, Boehringer Ingelheim, Boston Scientific, Bristol Myers Squibb, CSL Behring, Daiichi-Sankyo, DUKE Clinical Institute, Eli Lilly, Medtronic, Novartis, Terumo, and Vifor.

**Burkert M. Pieske** reports consulting fee from River Biomedics, payment or honoraria from Astra-Zeneca, Bayer, MSD, Novartis, and Boehringer Ingelheim, and minor shares in Imaging in Clinical Trials GmbH.

**Fausto J. Pinto** reports consulting fees and/or honoraria from Boehringher Ingelheim, Daichi Sankyo, Novartis, Servier, Vifor and Zydus and participation in the Advisory Board for Medtronic, Novartis, Servier, Vifor.

**Tienush Rassaf** has received speaker honoraria and consulting fees from AstraZeneca, Bayer, Pfizer, and Daiichi-Sankyo.

**Maurizio Volterrani** reported no conflict of interest.

**Stephan von Haehling** eports research support from Amgen, Boehringer Ingelheim, Pharmacosmos, IMI, and the German Center for Cardiovascular Research (DZHK).

**Markus S. Anker** reported no conflict of interest.

**Wolfram Doehner** has received consulting fees from Boehringer and personal fees from Aimediq, Bayer, Boehringer Ingelheim, Medtronic, Vifor Pharma and research support from EU (Horizon 2020), German ministry of Education and Research, German Center for Cardiovascular Research, Vifor Pharma, and ZS Pharma.

**Hüseyin Ince** reports being on data and safety monitoring board for RESHAPE-HF2.

**Friedrich Koehler** reports grants for Project 5G-MedCamp from German Federal Ministry of Economics and climate protection (BMWK) and grants for projects RESKRIVER and 6 G Health, consulting fees and/or payments or honoraria from BIOTRONIK, Boehringer-Ingelheim, Sanofi Germany GmbH, Novartis Germany (till 2022) and AMGEN Germany (in 2021).

**Gianluigi Savarese** has received grants and personal fees from Vifor, Boehringer Ingelheim, AstraZeneca, Novartis, Cytokinetics, and Pharmacosmos; personal fees from Servier, Medtronic, TEVA, Abbott, Edwards Lifesciences, INTAS, and Abbott; and grants from Boston Scientific, Merck, and Bayer, outside of the submitted work.

**Ursula Rauch–Kröhnert** reported no conflict of interest.

**Tommaso Gori** has received speaker fees and grant support from Abbott Vascular, Neovasc/Shockwave, Bristol-Myers Squibb/Pfizer, Bayer, Astra Zeneca, Novartis, Therox, SMT, and Insight Lifetech

**Teresa Trenkwalder** reports research grant from Pfizer unrelated to this work, consulting and lecture fees for Pfizer, Alnylam, Astra Zeneca, Bayer, Bristol Myers Squibb, Boehringer Ingelheim and Alexion, and travel support from Alnylam, Bayer, and Boehringer Ingelheim.

**Ibrahim Akin** reported no conflict of interest.

**Christina Paitazoglou** reported no conflict of interest.

**Iwona Kobielusz-Gembala** reported no conflict of interest.

**Witold Zmuda** reported no conflict of interest.

**Luca Kuthi** reported payment or honoraria from Boehringer Ingelheim and Novartis.

**Norbert Frey** reports receiving lecture fees/honoraria from AstraZeneca, Bayer Vital, Boehringer Ingelheim Pharma, Daiichi Sankyo, Novartis and Pfizer Pharma.

**Manuela Licka** reported no conflict of interest.

**Stefan Kääb** reported no conflict of interest.

**Karl-Ludwig Laugwitz** reported no conflict of interest.

**Piotr Ponikowski** has received consulting fees from Boehringer Ingelheim, AstraZeneca, Vifor Pharma, Amgen, Servier, Novartis, Bayer, MSD, Pfizer, Cibiem, Impulse Dynamics, Renal Guard Solutions, and BMS. PP has also received honoraria from Boehringer Ingelheim, AstraZeneca, Vifor Pharma, Amgen, Servier, Novartis, Berlin Chemie, Bayer, Pfizer, Impulse Dynamics, Renal Guard Solutions, BMS, and Abbott Vascular for lectures, presentations, speakers' bureaus, manuscript writing, or educational events.

**Mahir Karakas** is a part-time employee of 4TEEN4 Pharmaceuticals GmbH and reports grant and non-financial support from Adrenomed AG and CSL Vifor, as well as personal fees from Adrenomed AG, Sphingotec, Vifor, Daiichi-Sankyo, Pharmacosmos and 4TEEN4.
